# The Dadaab camps - Mitigating the effects of drought in the Horn (perspective)

**DOI:** 10.1371/currents.RRN1289

**Published:** 2011-12-15

**Authors:** Dr Osman Dar, Mishal Khan

**Affiliations:** ^*^Specialist Registrar Public Health, Institution: London School of Hygiene and Tropical Medicine, Location: London and ^†^Research Alliance for Advocacy and Development (RAAD)

## Abstract

Since mid 2011 the tragedy unfolding across the Horn of Africa following prolonged drought in the region has been a major focus for international relief operations and emergency aid. However, the most effective strategies for mitigating the effects of the drought have not been given sufficient media coverage or discussed critically enough in the public arena. Instead, while important and necessary, the focus has largely remained on emotive pleas for increased aid. This unfortunately, detracts from a considered discourse on the most effective interventions in the current circumstances and reduces scrutiny on performance of the primary agencies and bodies responsible for coordinating the relief effort. The authors present a personal perspective having recently returned from the Dadaab refugee camps where much of the relief effort has focused.

## The Dadaab camps

The Dadaab refugee camps, located in the North Eastern Province of Kenya approximately 60 miles from the Somalia/Kenya border and about 320 miles from the capital, Nairobi highlight concerns around the delivery and distribution of relief and aid. The three original camps, Ifo, Dagahaley and Hagadera were established in the early nineties following the onset of inter-clan conflict in Somalia. The vast majority of refugees present are Somalis from Central and Southern regions of Somalia. While the populations in the camps remained relatively stable from 1994 to December 2006, this year in particular has seen hugely increased numbers of refugees arriving over the border as a result of the drought. With over 160,000 new refugees having arrived in the first eleven months of 2011 alone, the camp complex now (as of November 2011) holds in excess of 460,000 refugees in total according to UNHCR - the primary agency mandated with coordinating relief activity within the camps.[1] In response to the current crisis, and after much negotiation, the Government of Kenya approved the opening of a fourth camp, Ifo-2, in an area of land between the Ifo and Dagahaley camps and a 5th camp, Kambioos south of Dadaab, close to camp Hagadera (see figure 1). Recent weeks, however, have seen a dramatic fall in the number of new arrivals with the registration of refugees completely suspended in the camps following the Kenyan military incursion into Somalia.[2]

## Current challenges

Using a combination of focus group discussions and one to one interviews with refugees and members of the host community and following detailed discussions of comprehensive assessments carried out by NGO service providers within and outside the camps the authors conducted a rapid needs assessment over July 2011. The following discussion draws from those results as well as from more recent assessments and developments in the months since. The paediatric age group continues to be the most badly affected in terms of severe acute malnutrition and global acute malnutrition rates amongst newly arriving refugees.[2] [3] [4] With constant anecdotal reports of children dying and being buried along the route to Dadaab from Somalia, sometimes within a few miles of Dadaab following days of walking, the location of new camps and service delivery centres was an issue of crucial importance.[4] [5] A recently concluded rapid assessment of mortality survey amongst new refugees conducted by the Centre for Disease Control (CDC) in the Dadaab camps during July and August similarly attested to this concern. [6] The study found a Crude Mortality Rate (CMR) of 1.94 deaths per 10,000 people per day (95% Confidence Interval 0.50-3.37) for the time period during their journeys versus a CMR of 0.86 (95% CI 0.57--1.15) prior to departure. One of the most effective interventions to reduce this continuing morbidity and mortality would have been to open smaller processing centres or camps on the border with Somalia at Liboi and other entry points and where refugees could have been registered and provided with initial care, water and food rations. Inflexibility in UNHCR regulations on the placement of new camps have in part been responsible for such facilities not being established. UNHCR site selection guidelines recommend refugees be “…settled at a reasonable distance from international borders as well as other potentially sensitive areas such as military installations.”; in practice this translates to at least 50 miles from an international border.[7] While security concerns for refugees and humanitarian workers given the conflict in Somalia and the associated rampant lawlessness are legitimate, the current movement of refugees is being driven primarily by drought and not the fighting. With many reports of deaths of children and rapes of women occurring en route to the camps within Kenya and the risks of refoulement (forcible repatriation of refugees by the host country) being small, a refugee centre at the border and smaller aid posts may well have been the best option for new refugees.[4] [8] The recently released International Organization for Migration report on migration mapping from Somalia to the Dadaab camps recommended establishing mobile water points to address the dehydration suffered by the refugees and pastoralists along the most common routes of entry, both active and passive disease surveillance during and after the migration process to curb the spread of disease amongst accompanying livestock and to address the rape and extortion of refugees through the establishment of a police task force in partnership with host communities along the pastoralist migratory routes.[9] While the publication of this report is welcome, its recommendations come over five months after the famine was officially declared in the region. Furthermore, the perceived risks to humanitarian aid workers from the establishment of border camps and outposts could have been mitigated by having them run and coordinated by strictly neutral agencies with the involvement of a mixture of international and local NGOs acceptable to both parties in the conflict in Somalia. The kidnap of two female Medicins Sans Frontiers aid workers from within the camps in October and the recent explosion of an Improvised Explosive Device in camp Hagadera highlights the ineffectiveness of simply trying to ensure security by concentrating all service delivery in the geographically confined area of the camp complex.

## Equity in service provision and equality in access

Another major area of concern in Dadaab is equity in the distribution of relief aid and service provision within refugee camps and adjacent areas. In the surrounding districts of Dadaab, Liboi and Fafi, the Host Community has also been suffering the consequences of the same drought plaguing the rest of the Horn. They, however, do not have equal access to services and relief routinely available to refugees. Healthcare services in the region exemplify these inequalities in aid provision. The three main camps are serviced by three hospitals with a total of 320 inpatient beds, 3 operating theatres, specialist outpatient services manned by doctors and visiting specialists. A structured referral system for specialized surgery and medical care in Garissa-the provincial capital, and Nairobi is also available to refugees. The Host Community which is approximately 150,000 strong and of a similar ethnic background to the majority of refugees only have access to outpatient health facilities within the camps. The only in-patient hospital service the Host Community has access to is a 30 bed district hospital (open to both the Host and refugee community) with 1 operating theatre. The most senior routine medical staff providing clinical care at this hospital are 2 clinical officers (not medically qualified doctors). A single Kenya Red Cross ambulance serves all three districts. These disparities are mirrored across other service sectors with old and new refugees having access to free food rations, water, sanitation and housing in an impoverished area where host communities are not routinely provided any of these services by the government or the NGOs.

## The city-state

A perversely functioning artificial city-state has thus developed supported entirely by international Non- Governmental Organizations (NGOs) where older refugee camp residents after years of free utilities and services have developed successful businesses and even rent out their refugee shelters to newer arrivals or Host Community members and who can undercut any Host Community business with lower overheads. Host Community Kenyans often register as refugees themselves in order to avail improved healthcare services and obtain rations when necessary. A jointly commissioned report by the Danish and Norwegian Embassies and the Government of Kenya in 2010 estimated that at least 40,500 (out of 150,000) host community members hold refugee ration cards.[10] In the light of these circumstances the incentive for eventual repatriation for refugees is minimal, and the drive for more people to arrive in an area of barren land not best equipped for naturally settling such large numbers of people is greater. Current abstractions at Dadaab from the Merti aquifer that provides the groundwater for the region have already lead to measurable reductions in both water quality and quantity locally.[11] While the stated aims of individual organizations operating in the Dadaab camps are firmly grounded in public health principle, the effects of focusing on their own individual spheres of operation and the lack of long term planning or an exit strategy has resulted in the increasingly unsustainable situation now developing. One reason for Kenya’s recently initiated military intervention into Somalia may well be to stem the flow of Somalis coming into the country and to the Dadaab camps in particular. Guidelines for the development of refugee camps in future should necessarily give due consideration to the resource and service circumstances of local host communities as well as incoming refugees. This will ensure that equitable provision based on identified need can be ensured comprehensively for both groups.

The long term key to resolving the situation in Dadaab lies in recognizing that pastoralist Somalis have ancient patterns of movement which involve crossing to and fro across the artificial line that divides Kenya and Somalia and that services designed to cater for this group should facilitate and take account of their nomadic lifestyle. NGOs, local or otherwise able to operate on both sides of the border should thus be supported in their efforts to provide this service. UNHCR itself should be encouraged to view the situation holistically and look beyond its remit of catering specifically to refugee needs by partnering with organizations with a wider mandate that includes the Host Community. This will ensure that Somalis that cross into the territory of Kenya are both willing and able to cross back into bordering Somali provinces and obtain equitably provided services there as well. 

## Acknowledgments

The authors would like to thank the team at Doctors WorldWide in Kenya for facilitating the field assignment to the Dadaab camps in July 2011. 

## Funding information

N/A 

## Competing interests

The authors have declared that no competing interests exist. 
